# A single site, multi-operator precision study for second-generation HR-pQCT

**DOI:** 10.1093/jbmrpl/ziag097

**Published:** 2026-06-01

**Authors:** Joshua R Stapleton, Nina Z Heilmann, Kristin Cattell, Andrew J Burghardt, Daniel P Beavers, Diana A Madrid, Kambrie M Brandt, S Delanie Lynch, Ashley A Weaver

**Affiliations:** Department of Biomedical Engineering, Wake Forest University School of Medicine, Winston-Salem, NC 27101, United States; Department of Gerontology and Geriatrics, Wake Forest University School of Medicine, Winston-Salem, NC 27101, United States; Department of Biomedical Engineering, Wake Forest University School of Medicine, Winston-Salem, NC 27101, United States; Department of Radiology and Biomedical Imaging, University of California San Francisco, San Francisco, CA 94158, United States; Department of Statistical Sciences, Wake Forest University, Winston-Salem, NC 27019, United States; Department of Biomedical Engineering, Wake Forest University School of Medicine, Winston-Salem, NC 27101, United States; Department of Biomedical Engineering, Wake Forest University School of Medicine, Winston-Salem, NC 27101, United States; Department of Biomedical Engineering, Wake Forest University School of Medicine, Winston-Salem, NC 27101, United States; Department of Biomedical Engineering, Wake Forest University School of Medicine, Winston-Salem, NC 27101, United States

**Keywords:** HR-pQCT, bone, BMD, bone microarchitecture, precision error, statistical methods

## Abstract

As HR-pQCT increases in popularity for studying bone dynamics, it becomes increasingly important to quantify the factors that impact rigor and reproducibility in research, particularly for longitudinal studies. Previously reported data for HR-pQCT precision primarily use first-generation HR-pQCT, and focus on single operators, with narrow study populations that reflect the population of a larger research question. This study at a single academic imaging center with standardized, single-analyst post-processing was designed to investigate how operator characteristics in scan acquisition and participant demographics influence measurement precision. In total, 45 adults (58% female; 22-82 yr; BMI 18.4-44.8 kg/m^2^) underwent same-day repeat HR-pQCT scanning of the distal tibia and radius, with 3 imaging technologists each acquiring 15 participant scan pairs, and registration was applied in post-processing to accurately depict the standard workflow at the center. The root-mean-square coefficient of variation was ≤1.3% for BMD measures and <2% for area measures in both the radius and tibia. Trabecular microarchitecture ranged from 1.22% to 2.24% in the radius and from 1.14% to 2.82% in the tibia, while cortical thickness precision was calculated as 1.99% in the radius and 1.57% in the tibia. Cortical porosity was much higher, at 24.66% in the radius and 20.90% in the tibia. Significant differences were not found when analyzed by technologists, or in statistical modeling of participant demographics as potential covariates. While final image quality scores varied among technologists, it did not influence precision outcomes. These results offer reference values for the least significant change and demonstrate that standardized technologist training can yield consistent scan-rescan precision across operators.

## Graphical Abstract

**Figure:**
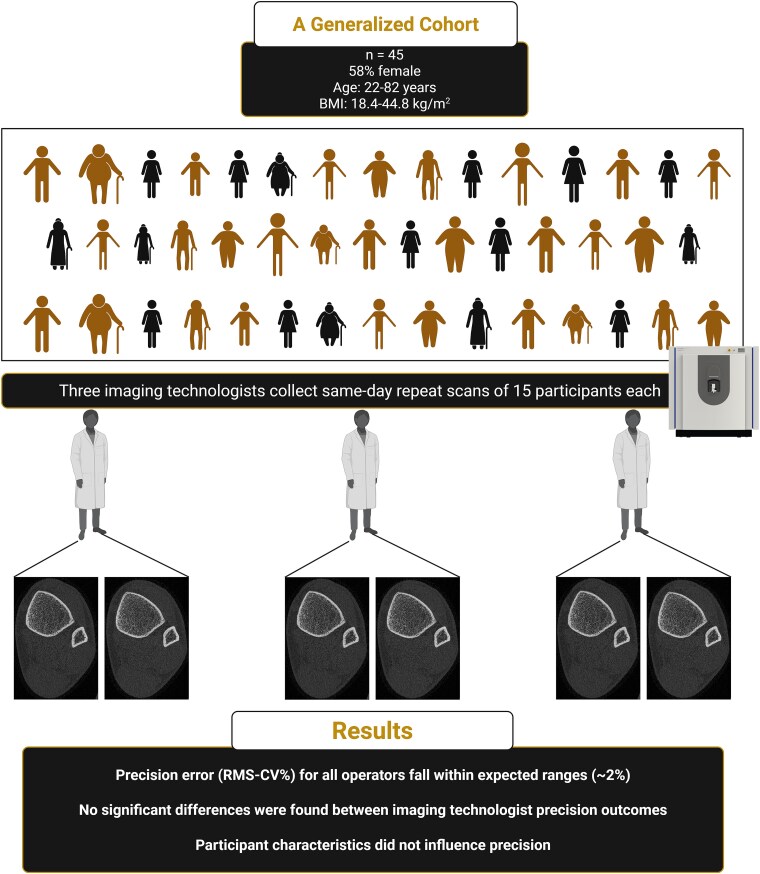

Created in Biorender. Stapleton, J. (2026) https://Biorender.com/a6t8kyc

## Introduction

HR-pQCT allows for the structural analysis of bone beyond clinical CT in vivo with resolutions under 100-μm, and separate characterization of the trabecular vs cortical bone compartments.^1^ Newer generations of HR-pQCT provide 61-μm voxel size, allowing for the direct measurement of bone microstructure, alongside density and strength measures through micro-finite element (μFE) models.^2^^,^^3^ The variety of measurements and unique insights provided by HR-pQCT are leading to wider adoption in research.

The emerging community has noted reproducibility as a key area of study for the advancement of HR-pQCT imaging, and more data are needed from new populations and imaging locations to aid in the understanding of factors that impact HR-pQCT reproducibility in research settings.^4–7^ The International Society for Clinical Densitometry (ISCD) recommends that precision studies be used to evaluate the least significant change (LSC) for densitometry measurements in longitudinal studies.^8^ A precision study allows for the quantification of variability in scanning, motion grading, and post-processing, as well as providing a measure of reproducibility of imaging that helps distinguish variability in data collection from true biological changes longitudinally.

Precision studies for both first- and second-generation HR-pQCT have been performed in a similar manner to precision studies done with DXA, typically using smaller sample sizes,^9^ within the population of an existing study,^10^ or focused on cortical metrics^11^ or multi-site precision.^12^^,^^13^ Often, multiple operators or scanning technologists are required when running multiple studies at a single center. Previous work has shown that standardized operator training reduces inter-operator variability of reference line positioning^7^; however, these analyses have yet to be extended to the second-generation HR-pQCT scanner, which offers a greater number of measured, rather than derived, metrics of bone quality.^14^

HR-pQCT precision studies are typically designed based on the ISCD precision study methodology guidelines, which require a minimum of 15 individuals scanned 3 times, or 30 individuals scanned twice.^8^^,^^9^ HR-pQCT precision studies reported in the literature typically draw samples directly from an ongoing cohort study or randomized controlled trial, allowing for the quantification of precision within that particular population.^4^^,^^13^^,^^15^^,^^16^ The nature of these studies, however, means that each study population requires a new precision study, which may or may not account for characteristics such as operator turnover or changes during the study timeframe. A single precision study, recruiting from a sample that is not limited to a specific cohort may, therefore, be supportive of research at multi-operator sites, and would reduce the burden on participants who may be receiving numerous other tests within the condensed timeframe of a study visit.

The root-mean-square coefficient of variation (RMS-CV%)^1^^,^^17^ describes differences in metrics acquired for short- and long-term precision estimates, which provides some robustness to data variation compared to the typical CV.^13^ Prior studies tended to use the non-dominant side per the standard HR-pQCT scan protocol;^1^ however, they varied by use of fixed^4^^,^^9^^,^^18^ or relative^10^^,^^13^^,^^15^^,^^16^ reference line offset procedures. Cortical porosity was noted to have considerably more variability in measurement when compared to other HR-pQCT derived parameters.^4^^,^^9^

Inter-operator variability remains a major contributor to HR-pQCT reproducibility beyond machine precision.^7^ In previous work by Chiba et al.^9^, an orthopedic surgeon with several years of experience collected and analyzed 3 sets of spaced (1+ week apart) HR-pQCT scans to quantify intra-operator precision in a cohort of 15 individuals aged 20-74 yr. Two additional orthopedic surgeons with variable prior exposure to HR-pQCT performed additional scans on the same participants to evaluate inter-operator precision, though a the standardized reference line placement did not appear to be used. The characterization of precision for these operators may not translate to a new scanning location, where imaging technologists with backgrounds in other modalities are trained using the standardized reference line protocol, but have minimal experience (<1 yr) with HR-pQCT at the time of testing. Previous work has shown that standardized training for operators and automation of the processing pipeline increase precision and reproducibility, particularly in the cortical compartment.^5^^,^^7^^,^^11^ Work by Whittier et al. measured differences in operator bone region extraction, but not in image collection, and showed that additional precision error may be introduced in the post-processing of images,^4^ and the standardization of the image analyst may therefore allow additional insight into the differences into the scan acquisition process. The distributed operator model may therefore reflect clinical research environments with work distributed across technologists of various imaging backgrounds.

Beyond machine precision, there may be human factors impacting final precision, predominantly through image quality and scan positioning.^19^ While scan positioning can be remedied through the use of image registration for common region extraction, motion artifact has been noted to impact the reproducibility of measurements. Factors impacting final image quality as well as the use of the standardized grading system should be investigated alongside the standard precision output.^19^^,^^20^ The initial image quality determined at scan time would be expected to have a consistent association with the final image quality of the fully reconstructed image and is important to investigate, as initial image quality is the standard guideline for determining whether a participant needs to be re-scanned in order to produce an image that can be included in the final dataset.^20^ Furthermore, participant characteristics, and particularly soft tissue, may also impact the scan-rescan precision.^21^^,^^22^ The operator, who has a notable impact on scan quality and scan-rescan precision, is also subject to many influences during scanning. While fatigue has not been directly studied as an influence on medical imaging quality, research in other fields has indicated that minimal repetitive activity can induce fatigue.^23^ Operators collecting many scans over the course of a day may be subject to fatigue within these tasks. The onset of “operator fatigue” may indirectly influence precision at no fault of a given subject, who happens to come later in the day vs earlier. These human characteristics should therefore also be investigated in the determination of precision, to aid in analysis and interpretation in populations with mixed characteristics.

In consideration of the preceding background, we conducted a study to investigate the precision and reproducibility of the Xtreme CT II (XCTII; Scanco Medical AG, Brüttisellen, Switzerland) scanner in the Wake Forest Baptist Medical Center (WFBMC) Translational Imaging Program (TIP). The TIP currently provides 3 technologists trained^7^ to collect HR-pQCT images. The collection of scans at both the radius and tibia locations, as well as basic demographic data such as age and body size will allow for the quantification of region-specific precision, as well as how variations in participant characteristics impact that precision.

The purpose of this study was to investigate the XCTII precision at a single clinical center with multiple scanning technologists with post processing performed by a single analyst. This study will support further research by quantifying the precision of the HR-pQCT measures being collected for prospective studies and randomized controlled trials.

## Materials and methods

### Study design

**Figure 1:**
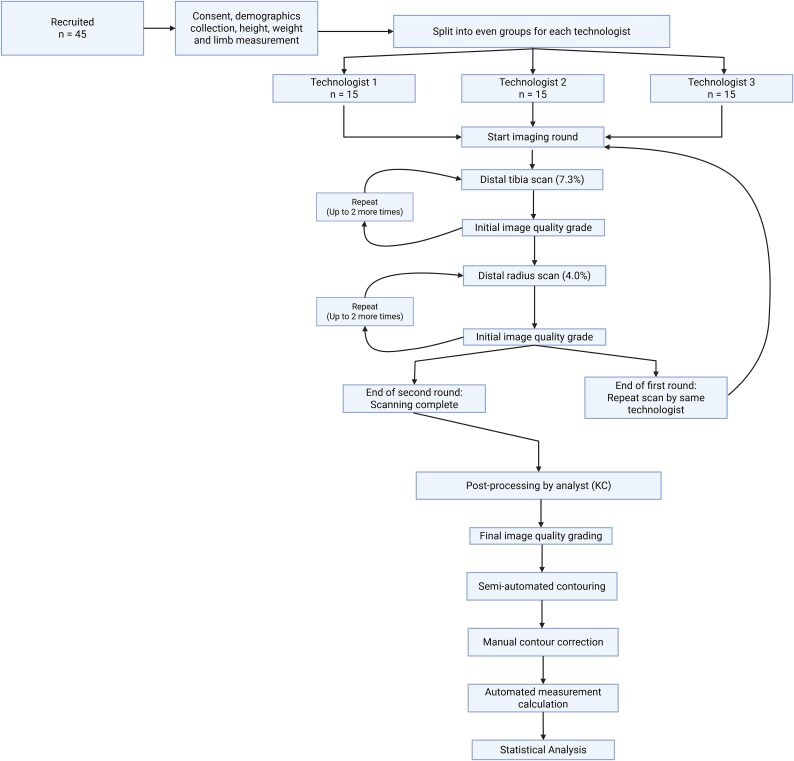
Diagram of precision study flow from recruitment to analysis. Created in BioRender. Weaver, A. (2026) https://BioRender.com/shmq6hp.

To accurately capture the reproducibility of the center, a similar number of HR-pQCT scans was collected by each technologist (Figure 1). Same-day repeat scans with repositioning were collected on a sample of 45 participants to allow sufficient scans to be acquired per operator (*n* = 15).^17^ This design meets the ISCD minimum number of scans, while allowing for both inter- and intra-operator variability assessments. Participants were recruited from the WFBMC and surrounding communities under Institutional Review Board (IRB) oversight (IRB00109036). An online form was used for initial screening of sex, age, and fracture history. Additional screening for contraindications (including hardware in the scan region and bone-active medications, pregnancy, pre-existing metabolic diseases or osteoporosis, history of fracture in the scan region, or having been non-weight bearing for >6-wk in the past year), and scheduling were completed over the phone. Written informed consent was collected from all participants. Data collected after consent included self-reported race/ethnicity, height measured with a stadiometer, weight measured with a calibrated digital scale, and tibia and radius limb lengths measured with a measuring tape (in mm). Scans were collected on the non-dominant limb, unless unilateral hardware or a fracture was present, in which case the unaffected limb was scanned. Imaging was scheduled in blocks of 4 participants, where each participant received XCTII scans of the distal radius and tibia, and then was scanned again by the same imaging technologist. Three imaging technologists (Crystal Duncan, Freda Crawford, and Wendy Baker) from the Wake Forest TIP program certified in X-ray imaging were trained using a standardized online protocol.^7^ These imaging technologists collected scans individually, and study staff were available for logistical assistance but did not influence image acquisition decisions.

### Image collection

XCTII images were collected using the standard manufacturer protocol (68 kVp, 1470 μA, 43 ms Integration Time, 14.0 cm field of view, 60.7 μm isotropic voxel size) with anatomically standardized regions using an offset based on the participant limb length, as measured by study staff.^6^ Single stack, 168 slice (10.2 mm) images were collected in the tibia and radius (7.3% and 4.0% relative offset respectively).^6^ Carbon fiber casts provided by the manufacturer were used to immobilize the limb during acquisition. Each technologist performed 3 study blocks with 4 participants each, and 1 study block with 3 participants each. Each imaging technologist performed reference line placement and initial image grading unassisted to more accurately reflect individual variation during collection. Initial image quality analysis followed the manufacturer suggested image grading scale for the fast, single slice reconstruction presented to operators immediately following scan collection (score 1-5 from no motion artifact to extreme motion artifact) where images of quality 3 or greater received an immediate in-session repeat scan in pursuit of higher quality, with up to 2 repeat scans per-location, per-imaging round for images with initial motion score grades of 3 or greater.^19^^,^^20^ Same-day repeat scans were then collected following the same protocol by the same technologist. Final image quality analysis was additionally performed on the fully reconstructed image by the analyst (K.C.), who did not influence grading of the initial image at scan time. Scans were registered using the manufacturer-provided 2D registration algorithm to correct for scan region offset per guideline recommendations for longitudinal studies.^1^ Results for distal bone parameters were presented after application of the 2D registration; however, μFE results were reported as unregistered due to standard registration procedures removing the perfectly parallel surfaces required on the anterior and posterior aspects of the scan for μFE analysis.

### Image post-processing for bone outcomes

Image post-processing was performed by a single analyst (K.C.) for final image quality grading and the manual correction of the standard manufacturer automated endosteal and endocortical contours. Manual correction of these measures was performed as needed to establish the boundaries of the outer bone structure, and the separation between cortical and trabecular compartments. Standard manufacturer software (IPL V6.0) was used to calculate the metrics presented in Table 1. Corrected contours were used to generate 3D μFE models in IPL V6.0 using standard homogeneous linear elastic material properties (10 GPa, Poisson’s ratio of 0.3), and loaded axially until 2% of all elements were strained beyond 0.7% following the Pistoia criterion.^24^ Post-collection grading was performed on the same scale as before, where subjects without 2 scans at a particular location with a final image quality <4 were excluded from that measurement.

**Table 1:** Measured HR-pQCT parameters.

Parameter	Abbreviation	Unit
**Total**		
**Total BMD**	Tt.BMD	mg HA/cm^3^
**Failure load (μFE)**	FL	kN
**Stiffness (μFE)**	–	kN/mm
**Cortical**		
**Cortical BMD**	Ct.BMD	mg HA/cm^3^
**Cortical area**	Ct.Ar	mm^2^
**Cortical thickness**	Ct.Th	mm
**Cortical porosity**	Ct.Po	%
**Trabecular**		
**Trabecular BMD**	Tb.BMD	mg HA/cm^3^
**Trabecular area**	Tb.Ar	mm^2^
**Trabecular bone volume fraction**	Tb.BV/TV	%
**Trabecular number**	Tb.N	1/mm
**Trabecular spacing**	Tb.Sp	mm
**Trabecular thickness**	Tb.Th	mm

### Statistical analysis

Study characteristics, including demographics and HR-pQCT bone parameters, were presented as mean ± SD and range (for continuous variables) or percentage (for categorical variables). Bland-Altman analyses were used to evaluate the agreement of data between the first and second measurements.

Precision was evaluated for each bone parameter at each skeletal site, considering participant characteristics including age, sex, and BMI, as well as technical factors such as scan technologist and study block position. Scan-rescan precision was quantified using the RMS-CV%, a standard metric in precision studies.^1^^,^^13^ The LSC, representing the minimal detectable difference at the 95% CI, was calculated as LSC = 2.77 × RMS-CV%.^1^^,^^13^

For each participant *i* and each bone parameter *j*, scan-rescan precision was defined as:


(1) \begin{eqnarray*} \mathrm{RMS}-\mathrm{CV}\%=\sqrt{\frac{{\left({X}_{i,j}^{(2)}-{X}_{i,j}^{(1)}\right)}^2}{{\left(\frac{X_{i,j}^{(1)}+{X}_{i,j}^{(2)}}{2}\right)}^2}}\times 100 \end{eqnarray*}

where ${X}_{i,j}^{(1)}$ and ${X}_{i,j}^{(2)}$ are the scan 1 and scan 2 measurements, respectively. Group-level RMS-CV% for parameter *j* was derived by aggregating participant-level estimates:


(2) \begin{eqnarray*} \mathrm{Group}\ \mathrm{RMS}-\mathrm{CV}\%=\sqrt{\frac{1}{N_j}\sum_{i=1}^{N_j}{\left(\mathrm{RMS}-\mathrm{CV}{\%}_{i,j}\right)}^2} \end{eqnarray*}

where *N_j_* is the number of participants contributing data for parameter *j*. This approach provides a summary of average variability while incorporating individual-level differences.

Group-level RMS-CV% was examined by BMI category, age category, scan technologist, and study block position (first-fourth). BMI was categorized as <25 kg/m^2^, 25-29.9 kg/m^2^, and ≥30 kg/m^2^ following World Health Organization cut points. Age was categorized both by quartiles and by clinically relevant cut-offs (<30 yr, 30-49 yr, 50-69 yr, ≥70 yr) chosen to reflect known phases of peak bone mass and age-related bone loss trajectories.^25^^,^^26^ Levene’s tests were used to assess homogeneity of variances, and Kruskal-Wallis tests were used to compare median RMS-CV% across categorical groups. To correct for multiple comparisons, *p*-values were adjusted using the Benjamini-Hochberg false discovery rate (FDR) procedure, ^27^ applied within predefined parameter families. These parameter families grouped related outcomes and were informed by previously described HR-pQCT parameter categories (Whittier et al.^1^). Specifically, parameters were categorized as density (BMD measures), porosity (Ct.Po), geometry (Ct.Ar, Tb.Ar, Tt.Ar, and Ct.Th), microarchitecture (Tb.BV/TV, Tb.N, Tb.Sp, and Tb.Th), and μFE (FL and stiffness).

To explore technical factors, precision was compared across scan technologists and study block positions. Additionally, the relationship between the number of repeat scans per session and final image quality was evaluated using Spearman rank correlations. Final image quality was also summarized as median (IQR) and compared across groups of 1, 2, or 3 scans using Kruskal-Wallis tests.

A linear mixed-effects model was applied to evaluate the associations of age, sex, and BMI with global precision across all HR-pQCT measurements (eqn (3)). For each participant (*i*) and parameter (*p*), RMS-CV% derived from duplicate scans was analyzed after log-transformation (log[RMS-CV%]) to reduce skewness and better satisfy model assumptions. Both continuous and categorical representations of age and BMI were considered. To account for systemic differences in precision across HR-pQCT parameters, the parameter type was included as a categorical fixed effect. A random intercept for participant (*u_i_*) was included to account for within-participant correlation arising from multiple measurements per individual, assuming an unstructured covariance. The acquiring technologist was not included as a fixed effect because each participant was scanned by a single operator, resulting in participants being nested within technologists.


(3) \begin{eqnarray*} {y}_{ip}={\beta}_0+{\beta}_1{\mathrm{Age}}_i+{\beta}_2{\mathrm{BMI}}_i+{\beta}_3{\mathrm{Sex}}_i+{\beta}_4{\mathrm{Parameter}\ \mathrm{Type}}_p+{u}_i+{\varepsilon}_{ip} \end{eqnarray*}

All analyses were performed using R (v4.5, R Core Team), where the density and microarchitectural metrics presented here are registered, and unregistered precision data are available in Table S1. Results from the μFE models are presented as unregistered, due to constraints of 2D registration leaving non-flat surfaces for standard μFE analysis.

## Results

### Study sample

The study sample (Table 2) had an age range of 22-82 yr, height of 142.2-188.0 cm and BMI of 18.4-44.8 kg/m^2^. A majority of participants were female (57.8%) and white (93.3%). Several participants (*n* = 1, 1 unusable distal tibia scan; *n* = 4, 1 unusable distal radius scan; *n* = 1, 2 unusable distal radius scans) were unable to produce an image pair with final image quality less than or equal to 3, and these scans were excluded from subsequent analysis. Following exclusion for image quality, the sample included 44 distal tibia and 40 distal radius scans.

**Table 2:** Study sample characteristics (*n* = 45).

Variable	Mean ± SD or %	Range
**Age (yr)**	45.2 ± 17.5	22-82
**Sex (% Female)**	57.8%	–
**Race (% White)**	93.3%	–
**Height (cm)**	169.6 ± 10.9	142.2-188.0
**Weight (kg)**	84.7 ± 21.2	42.6-145.6
**BMI (kg/m** ^**2**^ **)**	29.3 ± 6.2	18.4-44.8

### Precision estimation

**Figure 2:**
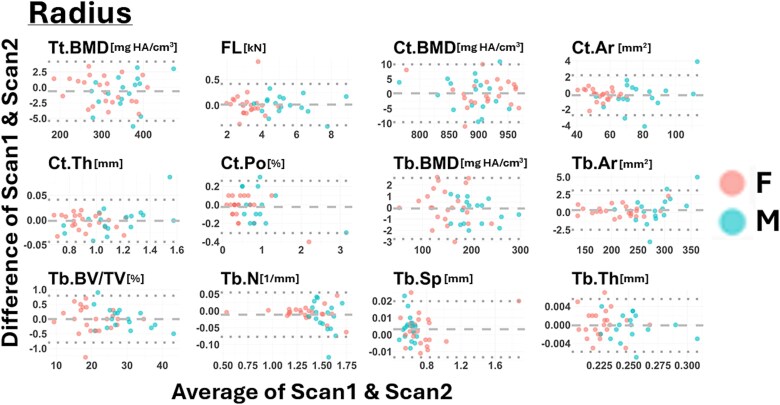
Bland-Altman plot for each HR-pQCT outcome for the distal radius colored by female and male.

**Figure 3:**
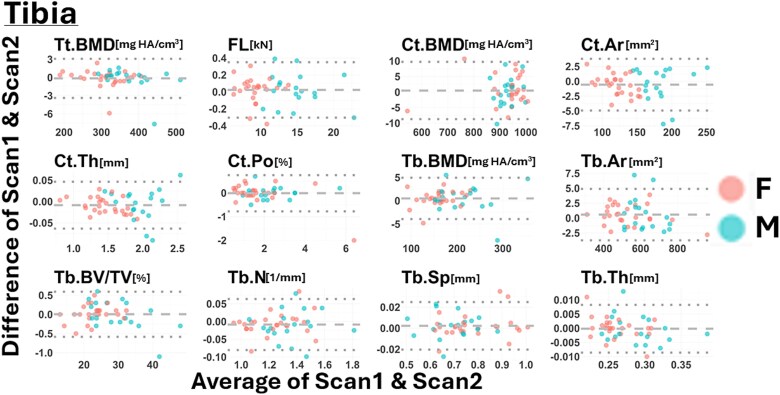
Bland-Altman plot for each HR-pQCT outcome for the distal tibia colored by female and male.

**Figure 4:**
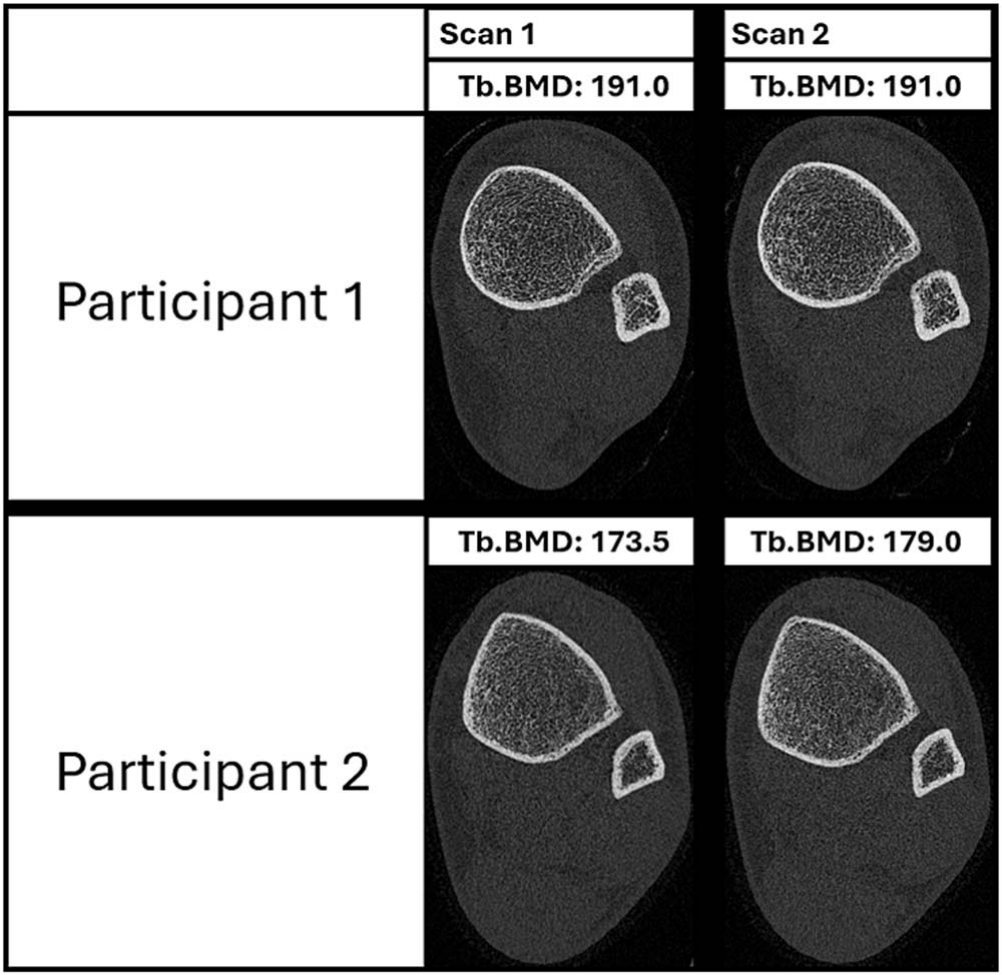
Comparison between participant with low (participant 1; 0.0) and high (participant 2; 5.5) scan-rescan differences for distal tibia trabecular BMD in mg HA/cm^3^.

Mean values of bone parameters from scan 1 and scan 2 and precision estimates using the previously described equations (eqn (1-2)) are presented in Table 3 for the radius and Table 4 for the tibia as RMS-CV% and LSC. Bland-Altman analysis for both the radius (Figure 2) and tibia (Figure 3) showed minimal bias and reasonable limits of agreement for all variables. Overall, precision estimates were highest (ie, worst) for cortical porosity and lowest (ie, best) for BMD and area measurements. Cortical metrics overall from the tibia had better precision estimates than the radius but did not appear to have better precision for trabecular microarchitecture. Visualization of differences between participants with higher and lower precision for BMD can be seen in Figure 4.

**Table 3:** Radius measures calculated in second-generation HR-pQCT.

Parameter	Physical units		Precision	
	Scan 1 (Mean ± SD)	Scan 2 (Mean ± SD)	RMS-CV%	LSC (%)
**Total**				
**Tt.BMD (mg HA/cm**^**3**^**)**	325.18 ± 60.14	324.17 ± 59.92	0.77	2.13
**FL (kN)**	4.19 ± 1.55	4.07 ± 1.51	5.37	14.86
**Stiffness (kN / mm)**	77.67 ± 29.31	75.60 ± 28.65	4.93	13.65
**Cortical**				
**Ct.BMD (mg HA/cm**^**3**^**)**	899.73 ± 44.93	903.07 ± 45.71	0.55	1.53
**Ct.Ar (mm**^**2**^**)**	65.44 ± 16.55	64.01 ± 16.86	1.79	4.95
**Ct.Th (mm)**	1.02 ± 0.21	1.01 ± 0.21	1.99	5.51
**Ct.Po (%)**	0.70 ± 0.59	0.64 ± 0.54	24.66	68.32
**Trabecular**				
**Tb.BMD (mg HA/cm**^**3**^**)**	173.76 ± 49.27	170.59 ± 48.69	0.86	2.38
**Tb.Ar (mm**^**2**^**)**	247.30 ± 57.27	240.24 ± 56.28	0.51	1.42
**Tb.BV/TV (%)**	24.06 ± 7.37	23.60 ± 7.23	2.05	5.69
**Tb.N (1/mm)**	1.41 ± 0.23	1.39 ± 0.22	2.24	6.21
**Tb.Sp (mm)**	0.68 ± 0.22	0.69 ± 0.22	1.38	3.84
**Tb.Th (mm)**	0.24 ± 0.02	0.24 ± 0.02	1.22	3.39

Abbrevations: Ct.Ar, cortical area; Ct.BMD, cortical BMD; Ct.Th, cortical thickness; Ct.Po, cortical porosity; FL, Failure load; LSC, least significant change; RMS-CV%, root-mean-square coefficient of variation; SD, standard deviation; Tb.BMD, trabecular BMD; Tb.Ar, trabecular area; Tb.BV/TV, trabecular bone volume fraction; Tb.N, trabecular number; Tb.Sp, trabecular spacing; Tb.Th, trabecular thickness; Tt.BMD, total BMD.

**Table 4:** Tibia measures calculated in second-generation HR-pQCT.

Parameter	Physical units		Precision	
	Scan 1 (Mean ± SD)	Scan 2 (Mean ± SD)	RMS-CV%	LSC (%)
**Total**				
**Tt.BMD (mg HA/cm**^**3**^**)**	336.35 ± 66.46	336.21 ± 66.05	0.45	1.25
**FL (kN)**	11.55 ± 3.79	11.58 ± 3.78	1.55	4.30
**Stiffness (kN/mm)**	215.28 ± 73.46	215.70 ± 72.99	1.72	4.76
**Cortical**				
**Ct.BMD (mg HA/cm**^**3**^**)**	928.06 ± 74.28	928.55 ± 74.95	0.53	1.25
**Ct.Ar (mm**^**2**^**)**	145.98 ± 38.44	145.41 ± 38.22	1.51	4.19
**Ct.Th (mm)**	1.66 ± 0.37	1.65 ± 0.37	1.57	4.35
**Ct.Po (%)**	1.95 ± 1.35	1.94 ± 1.20	20.90	57.89
**Trabecular**				
**Tb.BMD (mg HA/cm**^**3**^**)**	182.30 ± 50.53	182.79 ± 50.58	1.26	3.49
**Tb.Ar (mm**^**2**^**)**	563.46 ± 124.87	564.03 ± 124.44	0.40	1.10
**Tb.BV/TV (%)**	25.86 ± 6.99	25.86 ± 6.92	1.14	3.16
**Tb.N (1/mm)**	1.29 ± 0.18	1.28 ± 0.19	2.82	7.82
**Tb.Sp (mm)**	0.74 ± 0.12	0.75 ± 0.12	1.60	4.44
**Tb.Th (mm)**	0.27 ± 0.03	0.27 ± 0.03	1.60	4.44

Abbrevations: Ct.Ar, cortical area; Ct.BMD, cortical BMD; Ct.Th, cortical thickness; Ct.Po, cortical porosity; FL, Failure load; LSC, least significant change; RMS-CV%, root-mean-square coefficient of variation; SD, standard deviation; Tb.BMD, trabecular BMD; Tb.Ar, trabecular area; Tb.BV/TV, trabecular bone volume fraction; Tb.N, trabecular number; Tb.Sp, trabecular spacing; Tb.Th, trabecular thickness; Tt.BMD, total BMD .

Precision estimation on a per-technologist basis is presented in Table S2 as just RMS-CV%. Based on Levene’s and Kruskal-Wallis tests comparing RMS-CV%, there are no statistically significant differences in variance or median, respectively, between the technologists (all *p* > .05). Temporal analysis was performed to assess whether precision varied based on the order of the participant in the scan session (Table S3). Radius cortical area, and tibia trabecular area, trabecular thickness, and failure load measures showed significant differences in median precision across positions within the study block (Kruskal-Wallis tests), but these differences did not follow a consistent directional trend indicative of increasing precision error over time. Additionally, Levene’s test indicated significant differences in variability only for tibial trabecular area, failure load, and stiffness. Following FDR correction, only failure load and stiffness remained statistically significant.

Some differences in RMS-CV% were seen by sex for trabecular bone at the radius and tibia, with slightly higher (worse) precision estimates among males with the exception of radial Tb.BMD (Table 5). Assessment of precision by BMI category (Table S4) showed no differences in median precision (RMS-CV%) or variance between individuals with BMI <25, 25 to <30 and ≥30 kg/m^2^. Assessment by age group (Table S5) yielded no differences in median or variance of precision estimates across age categories.

**Table 5:** Precision estimate (RMS-CV%) by sex.

	Distal radius		Distal tibia	
Sex	Female *n* = 23	Male *n* = 17	Female *n* = 26	Male *n* = 18
**Total**				
**Tt.BMD (mg HA/cm**^**3**^**)**	0.75	0.78	0.45	0.45
**FL (kN)**	6.49	3.27	1.65	1.40
**Stiffness (kN/mm)**	5.94	3.06	1.79	1.61
**Cortical**				
**Ct.BMD (mg HA/cm**^**3**^**)**	0.51	0.61	0.55	0.50
**Ct.Ar (mm**^**2**^**)**	1.41	2.20	1.51	1.51
**Ct.Th (mm)**	1.74	2.28	1.39	1.81
**Ct.Po (%)**	27.30	20.56	23.93	15.50
**Trabecular**				
**Tb.BMD**^**a**^ **(mg HA/cm**^**3**^**)**	**1.02**	**0.57**	1.27	1.24
**Tb.Ar (mm**^**2**^**)**	0.37	0.66	0.34	0.47
**Tb.BV/TV**^**b**^ **(%)**	2.39	1.47	**1.07**	**1.23**
**Tb.N**^**a**^^**,**^^**b**^ **(1/mm)**	**1.33**	**3.07**	2.51	3.22
**Tb.Sp**^**b**^ **(mm)**	1.30	1.49	**1.40**	**1.85**
**Tb.Th**^**b**^ **(mm)**	1.29	1.13	**1.48**	**1.76**

^a^ *p* < .05 from Levene’s test comparing variance in precision values between sexes, with significant values bolded.

^b^ *p* < .05 from Kruskal-Wallis test comparing median precision values between sexes, with significant values bolded.

All *p* > .05 from Kruskal-Wallis and Levene’s tests comparing variance in precision values between sex following false discovery rate correction.

Abbrevations: Ct.Ar, cortical area; Ct.BMD, cortical BMD; Ct.Th, cortical thickness; Ct.Po, cortical porosity; FL, Failure load; LSC, least significant change; RMS-CV%, root-mean-square coefficient of variation; SD, standard deviation; Tb.BMD, trabecular BMD; Tb.Ar, trabecular area; Tb.BV/TV, trabecular bone volume fraction; Tb.N, trabecular number; Tb.Sp, trabecular spacing; Tb.Th, trabecular thickness; Tt.BMD, total BMD.

Linear mixed effects models revealed no consistent associations of age, BMI, or sex with precision metrics (Table 6). Parameter type was significantly associated with RMS-CV% with density measures serving as the reference category. Compared with density, RMS-CV% was higher for porosity (beta = 398%), geometry (22.2%), microarchitecture (35.4%), and μFE (73.2%) indicating systematic differences in precision across parameter classes and no consistent associations with demographic factors.

**Table 6:** Linear mixed-effects models for global HR-pQCT precision results for distal bone sites using clinically relevant age and BMI categories.

	Coefficient (RMS-CV%)	*p*-value
**Age < 30**	*Ref*	
**Age 30-49**	1.30	.86
**Age 50-69**	9.32	.22
**Age 70+**	10.3	.35
**Normal/Underweight (BMI < 25)**	*Ref*	
**Overweight (25 ≤ BMI < 30)**	−7.66	.79
**Obese (BMI ≥ 30)**	−1.96	.27
**Female sex**	*Ref*	
**Male sex**	6.11	.31
**Parameter types** ^**a**^		
**Density**	*Ref*	
**Porosity**	398.4	**<.001**
**Geometry**	22.2	**<.001**
**Microarchitecture**	35.4	**<.001**
**μFE**	73.2	**<.001**

^a^Parameter types: density (vBMD measures); porosity (Ct. Po); geometry (Ct. Ar, Tb.Ar, Tt.Ar, Ct.Th); microarchitecture (Tb.BV/TV, Tb.N, Tb.Sp, Tb.Th); μFE (FL, Stiffness).

Statistically significant p-values <.05 are bolded. Abbrevations: BMI, body mass index; Ct.Ar, cortical area; Ct.BMD, cortical BMD; Ct.Th, cortical thickness; Ct.Po, cortical porosity; FL, Failure load; LSC, least significant change; μFE, micro-finite element; RMS-CV%, root-mean-square coefficient of variation; Tb.BMD, trabecular BMD; Tb.Ar, trabecular area; Tb.BV/TV, trabecular bone volume fraction; Tb.N, trabecular number; Tb.Sp, trabecular spacing; Tb.Th, trabecular thickness; Ref, reference.

### Image quality analysis

**Figure 5:**
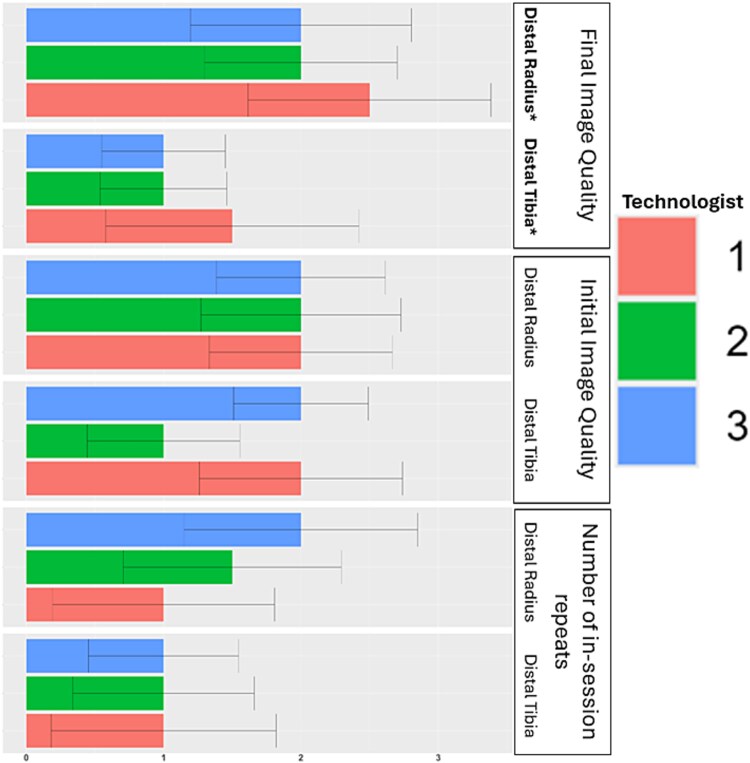
Scan quality and number of scans collected on a per technologist basis. Bolded *p** < .05** from Kruskal-Wallis test comparing median values between technologists.

Image quality assessment for initial acquisition (initial grading for motion artifact used to determine if repeat scan is necessary^20^) and final evaluation during the scan post-processing (ie, before and after full reconstruction) is shown in Figure 5. Initial image quality grading was not significantly different between technologists; however, the final image quality grading was significantly different. The number of in-session repeats due to motion artifacts present in the initial image was not different between technologists. The average number of repeats per session for the radius (mean ± SD; 1.8 ± 0.84) and tibia (1.4 ± 0.69) was below the maximum allowable in-session repeats, indicating that the full number of scans was often not needed.

## Discussion

This precision study expanded upon the current body of literature reporting precision estimates for second-generation HR-pQCT. We collected same-day repeat scans from 45 individuals across a spectrum of demographics, with 3 different technologists from various imaging backgrounds (MRI, CT, and PET) and with minimal previous HR-pQCT experience. Each operator collected same-day repeat scans on a unique cohort of 15 individuals. These operators had been trained using standardized methods for reference line placement; however, they did not have extended experience in patient positioning and scan collection. A single analyst performed all post-processing to provide an estimation of precision localized to scan acquisition. Secondary analyses were intended to explore factors influencing precision, though they should not be interpreted as deterministic due to the moderate sample size and power. Our protocol implemented routine in-session repeats when initial scans exceeded the standard re-scan threshold and implemented 2D registration for density and microarchitectural outcomes to mirror longitudinal workflows. Overall, we found good precision estimates, with measures of density shown to be more reliable than microarchitecture, and minimal inter-technologist difference in small subgroup analyses.

Consistent with prior reports, the RMS-CV% precision estimates for BMD and area were generally <1%-2% (0.4%-2.4%, 0.4%-3.3%, reported respectively).^1^ Microarchitecture precision measurements tended to be slightly higher than BMD and area, however, still fell within the generally expected ranges reported for the XCTII of 1%-2%.^1^ Cortical porosity had a very large precision error in our cohort at both the radius and the tibia—roughly double the expected error of ~10%, however, should be interpreted with caution due to low mean values causing distortion of CV precision estimates.^1^ The Bland-Altman analysis indicated a minimal bias (0.008%, −0.022%; tibia and radius, respectively) with relatively narrow upper (0.753%, 0.260%; tibia and radius, respectively) and lower (−0.776%, −0.305%; tibia and radius, respectively) limits of agreement. Precision of the cortical measures in the tibia were observed to be slightly lower than in the radius, but trabecular measurements were not. This coincides with higher absolute values such as area, thickness and BMD that may indicate the overall thinner cortex of the radius plays a role in worse precision values, as smaller areas may be more prone to error with the consistently higher motion artifacts in the radius.^28^ Comparison of these results against prior inter-operator precision estimates shows that while standardized reference line training and registration are important for studies to implement, there may still be a component of experience which should be adequately trained prior to the conduction of clinical research. Notably, the selection for the primary outcome should be carefully considered, and if measurements of microarchitecture are of interest, then great care should be taken to ensure consistency across measurements and that consistent training be applied for longitudinal work.^7^

Failure load and stiffness from μFE measurements are reported using unregistered data, as the IPL FE solver requires parallel endplates.^1^ Precision error in the radius was far greater than in the tibia; however, both locations appeared to be higher on average than the expected ~1% RMS-CV% for short-term μFE. ^1^ Worse precision error of these unregistered results likely corresponds to differences in reference line placement leading to reduction of reliability in these measurements. In consideration of this, studies which wish to employ μFE measures as a primary outcome should consider rigorous operator training and familiarization with scanning protocols beyond the in-person training provided by Scanco prior to scan collection within and across sites. Furthermore, employment of 3D registration may allow for common volume regions to be extracted while maintaining μFE capability, though this work has not moved into standard practice yet.^29–31^

Though not the primary focus of the study, we attempted to characterize inter-technologist variability by examining the characteristics of precision, scan grading, and repeat acquisitions. This study was not powered or designed to detect differences between technologists, as each technologist acquired same-day repeat scans on a unique cohort of individuals, and therefore, a direct comparison is not possible in this dataset. While results should be interpreted with caution due to lower sample size when splitting the cohort into groups, we did not see any differences in precision estimates between technologists. This is a positive result and supports that standardized training increases the reliability of scanning, and is in-line with previous reports characterizing inter-technologist precision to be acceptable.^7^^,^^9^ Image quality analysis indicated that technologists produce similar estimation for initial image quality when assessing for rescan; however, the analyst-determined final image quality during post-processing shows differences in both the radius and tibia. Variation in final image quality, but not initial image quality, indicates that final image quality may differ by technologist for images with the same initial grade provided during scanning. This is not a definitive test for differences in image quality grading, as the technologists analyzed different images; however, we nonetheless attempted to characterize differences in technologist grading criteria. In light of the lack of differences in the calculated precision estimates between technologists, it can reasonably be determined that differences in initial image quality assessment do not appear to affect the final measures drastically. This conclusion, however, may only hold true when the initial image quality is less than the repeat threshold based on the guidelines, as our per-technologist median image quality was under 3 for all technologists (Figure 5) and images of quality >3 were excluded from precision analyses. This discrepancy does perhaps warrant consideration of having multiple operators inspect a consistent set of images to ensure that all provide a similar grading of image quality.

While we hypothesized that long scanning blocks may induce “operator fatigue”, we did not find an association between the quality of scans at the end of a scanning session and quality of scans towards the beginning. Both the cortical and trabecular areas saw differences in median image quality; however, there was not a consistent increasing trend to indicate that image quality decreased based on position in the scan block. From these results, we can assert that scheduling multiple (at least up to 8 in total) scans back-to-back does not impact the HR-pQCT measurement precision.

Across the age, sex, and BMI strata, we observed no consistent associations with precision in parameter-specific analyses, or in mixed-effect models that accounted for within-participant correlation and parameter-type differences in reproducibility. The pooled mixed-effects model attempted to discern whether, on average, certain individual characteristics impact precision across HR-pQCT outcomes, rather than assess individually measured parameters. Furthermore, categorization into clinically relevant subgroups allowed for the analysis of potentially non-linear relationships between participant characteristics and bone measures. Participant characteristics did not have significant effects on precision in our cohort, and instead we saw that variation in precision error is greater between measured parameters than between subjects. From this, we can consider that demographics may have less importance than the parameter type when selecting outcomes for clinical studies; however, a larger cohort would be required to fully confirm the impact, or lack thereof, of subject characteristics on HR-pQCT imaging reproducibility.

Participant BMI and age did not appear to be associated with precision, as no differences were seen across the BMI or age categories in this study. Additionally, mixed models did not show an association between age and precision; however, the older age groups did show a trend towards worse precision estimates. A review of previous work notes a consistent decline in bone microstructure with age for both men and women,^32^ and the reduced size of the microstructure may result in worse precision either due to increased variability within the surrounding scan region or worse registration results from reduced high-contrast bone. It is possible that the increasing precision error estimates with age are not fully fleshed out due to the reduced and small sample size of the 50-59 (*n* = 11 radius, *n* = 13 tibia) and 70+ (*n* = 4) as opposed to the relatively larger sample size in younger groups (<30, *n* = 14; 30-49, *n* = 13). In the stratified analysis, we saw some indication that increasing BMI may play a role in scan-rescan precision, particularly in the tibia. This may be due to scattering artifacts produced by higher levels of soft tissue in the scan region, which may influence reproducibility at the machine, rather than the biological or human factor level (ie, scan positioning, reference line placement).^21^^,^^22^ Future studies could extract precision results from this study for their specific participant demographic, rather than from our whole precision study sample, to use for estimation of the least-significant change during longitudinal studies.

Based on our findings, the data presented in this study support longitudinal studies, which may make use of multiple scan collectors. Though limited by lack of true comparators, we found that operators achieved similar precision estimates on unique populations. Some minor differences, perhaps influenced by population or other non-controlled external factors, were found between operator image grading. Additionally, we saw major improvements with the application of standard 2D registration to our results. This indicates that the application of standardization protocols is important for characterizing longitudinal cohorts, but also that operator agreement is key for creating reproducible images. Standardized training is incredibly important, but at a site with multiple operators collecting images, it perhaps is more important to develop dialog between scan collectors to ensure agreement between individuals.

Overall, the strengths of this study include a population that encompasses a range of body shapes and sizes which will therefore support a range of study populations and was performed in accordance with guideline-recommended protocols and training.^1^ We implemented the use of relative offset landmark and scanning protocols for the distal tibia and radius. For precision outcomes, we calculated RMS-CV% and LSC for reporting with 2D registration applied when appropriate. Image pairs lacking 2 scans with acceptable final motion grades (>3) were excluded from analysis; however, Table S6 contains the differences seen with the inclusion/exclusion of low-quality images. Though some images were excluded from our final analysis, we found that on average our participants did not require the maximum number of allowable in-session repeats. In contrast to guidelines, however, in accordance with what may be more realistic at larger clinical centers, we use multiple scanning technologists to collect scans in order to present what short-term reproducibility is like for a site conducting studies with a team of new technologists performing image collection. Some limitations of this study should be considered for the application of our results. Firstly, the study population is predominantly White and therefore fails to be fully generalizable. Further work should push to expand precision data in a broader population to ensure that diverse study populations are fully supported. Additionally, some quantification of true inter-technologist precision is lacking, as all scan-rescan sessions were performed by the same technologist, and no subject therefore received scans by multiple technologists. Instead, we rely on lack of differences in precision outcomes to determine that there is not a significant difference between technologists in precision error. The sample size is further limited through the exclusion of individuals with unacceptable final motion artifact (grade > 3). As indicated in Table S6, the exclusion of these individuals does, in most cases, positively bias our results, however, per general recommendations, the exclusion of these individuals should be followed for both cross-sectional and longitudinal analyses. The limitations of this study can be remedied through continued investigation of HR-pQCT precision by further recruitment and cohort expansion.

## Conclusion

Overall, this precision study will support future projects that rely on HR-pQCT to investigate changes in bone density over time. The precision estimates for density (0.45%-1.26%), area (0.40%-1.79%), trabecular microarchitecture (1.14%-2.82%), cortical thickness (1.57%-1.99%), and μFE failure load (1.55%-5.37%) were acceptable for both the distal radius and tibia for use in longitudinal work. Additionally, our examination of demographic, operator-related and the possibility of fatigue will help inform the study design for future research. While some differences were seen with subject demographics such as sex, a larger sample size is required to fully elucidate these associations. Overall, we conclude that the inclusion of standardized training for HR-pQCT operators allows for flexibility of the scanning operator in longitudinal studies.

## Supplementary Material

PrecisionStudyPaper_Supplemental_ziag097

## Data Availability

To maintain the privacy of study participants, the data underlying these analyses are not publicly available at this time. Data may be shared upon reasonable request to the authors.
