# Supplementary material for: A single site, multi-operator precision study for second-generation HR-pQCT
Source: JBMR Plus. 2026 Jun 1;10(7):ziag097. doi: 10.1093/jbmrpl/ziag097 (PMC13283432; doi:10.1093/jbmrpl/ziag097)
Supplement: PrecisionStudyPaper_Supplemental_ziag097 [file precisionstudypaper_supplemental_ziag097.docx]

Supplemental Table 1: Unregistered precision results for precision cohort

| **Parameter** | **Distal Radius (n=40)** | | | | **Distal Tibia (n=44)** | |  |  |
| --- | --- | --- | --- | --- | --- | --- | --- | --- |
|  | Scan 1  [Mean ± SD] | Scan 2  [Mean ± SD] | **RMS-CV%** | **LSC** | Scan 1  [Mean ± SD] | Scan 2  [Mean ± SD] | **RMS-CV%** | **LSC** |
| **Total** |  |  |  |  |  |  |  |  |
| Tt.BMD [mg HA/cm^3^] | 324.85±60.23 | 322.10±61.82 | 6.83 | 18.93 | 336.07±67.05 | 335.94±65.62 | 2.65 | 7.35 |
| FL [kN] | 4.09±1.55 | 4.09±1.52 | 5.89 | 16.30 | 11.65±381 | 11.68±3.79 | 1.54 | 4.25 |
| Stiffness [kN/mm] | 75.8±29.30 | 75.84±28.69 | 5.48 | 15.17 | 217.21±73.76 | 217.62±73.31 | 1.70 | 4.71 |
| **Cortical** |  |  |  |  |  |  |  |  |
| Ct.BMD [mg HA/cm^3^] | 899.60±49.76 | 900.76±44.75 | 3.00 | 8.30 | 927.77±74.97 | 929.10±74.47 | 1.12 | 3.11 |
| Ct.Ar [mm^2^] | 65.25±16.58 | 63.69±16.89 | 4.84 | 13.42 | 145.70±38.65 | 145.18±37.98 | 2.90 | 8.04 |
| Ct.Th [mm] | 1.02±0.21 | 1.01±0.22 | 9.11 | 25.25 | 1.66±0.38 | 1.65±0.37 | 4.57 | 12.66 |
| Ct.Po [%] | 0.69±0.58 | 0.63±0.55 | 26.38 | 73.06 | 1.95±1.40 | 1.95±1.18 | 16.42 | 45.48 |
| **Trabecular** |  |  |  |  |  |  |  |  |
| Tb.BMD [mg HA/cm^3^] | 173.58±48.13 | 170.70±48.69 | 3.22 | 8.92 | 182.52±50.39 | 182.90±50.59 | 2.44 | 6.75 |
| Tb.Ar [mm^2^] | 248.77±59.57 | 243.01±58.26 | 10.28 | 28.46 | 564.61±125.44 | 565.30±124.92 | 4.82 | 13.35 |
| Tb.BV/TV [%] | 24.02±7.19 | 23.62±7.22 | 4.38 | 12.12 | 25.89±6.97 | 25.87±6.92 | 2.35 | 6.51 |
| Tb.N [1/mm] | 1.41±0.23 | 1.39±0.22 | 3.48 | 9.63 | 1.29±0.18 | 1.28±0.19 | 3.26 | 9.04 |
| Tb.Sp [mm] | 0.68±0.21 | 0.69±0.23 | 3.19 | 8.85 | 0.74±0.12 | 0.74±0.12 | 2.90 | 8.03 |
| Tb.Th [mm] | 0.24±0.02 | 0.24±0.02 | 1.59 | 4.40 | 0.27±0.03 | 0.27±0.03 | 1.76 | 4.87 |

**Note:** µFE results are always unregistered, due to constraints of the standard registration analysis leaving non-parallel surfaces

Supplemental Table 2: Precision results (RMS-CV%) for registered data split by technologist.

| **Parameter** | **Distal Radius (n=40)** | | | **Distal Tibia (n=44)** | | |
| --- | --- | --- | --- | --- | --- | --- |
|  | Tech 1 | Tech 2 | Tech 3 | Tech 1 | Tech 2 | Tech 3 |
| **Total** |  |  |  |  |  |  |
| Tt.BMD [mg HA/cm^3^] | 0.70 | 0.76 | 0.82 | 0.70 | 0.29 | 0.23 |
| FL [kN] | 6.23 | 3.07 | 6.45 | 1.72 | 1.43 | 1.50 |
| Stiffness [kN/mm] | 6.01 | 2.76 | 5.74 | 1.80 | 1.65 | 1.72 |
| **Cortical** |  |  |  |  |  |  |
| Ct.BMD [mg HA/cm^3^] | 0.62 | 0.40 | 0.64 | 0.39 | 0.61 | 0.57 |
| Ct.Ar [mm^2^] | 1.44 | 1.82 | 1.99 | 0.94 | 1.89 | 1.52 |
| Ct.Th [mm] | 2.22 | 1.57 | 2.19 | 1.23 | 2.05 | 1.29 |
| Ct.Po [%] | 27.18 | 26.78 | 19.73 | 18.07 | 20.97 | 23.17 |
| **Trabecular** |  |  |  |  |  |  |
| Tb.BMD [mg HA/cm^3^] | 0.88 | 0.85 | 0.85 | 1.48 | 1.44 | 0.72 |
| Tb.Ar [mm^2^] | 0.59 | 0.50 | 0.45 | 0.28 | 0.52 | 0.34 |
| Tb.BV/TV [%] | 2.56 | 1.69 | 1.96 | 1.38 | 1.13 | 0.87 |
| Tb.N [1/mm] | 3.21 | 1.35 | 2.08 | 2.43 | 2.99 | 2.99 |
| Tb.Sp [mm] | 2.10 | 0.78 | 1.17 | 1.29 | 1.74 | 1.71 |
| Tb.Th [mm] | 1.37 | 1.05 | 1.28 | 1.48 | 2.04 | 1.15 |

**Note:** All p>0.05 from Kruskal-Wallis and Levene’s tests comparing variance in precision values between technologists

Supplemental Table 3: Precision results (RMS-CV%) for registered data separated by position in study scan block.

|  | **Distal Radius (n=40)** | | | | **Distal Tibia (n=44)** | | | |
| --- | --- | --- | --- | --- | --- | --- | --- | --- |
| Position in Study Block | 1 | 2 | 3 | 4 | 1 | 2 | 3 | 4 |
|  | n=9 | n=11 | n=11 | n=9 | n=12 | n=11 | n=12 | n=9 |
| **Total** |  |  |  |  |  |  |  |  |
| Tt.BMD [mg HA/cm^3^] | 0.86 | 0.69 | 0.78 | 0.74 | 0.61 | 0.21 | 0.20 | 0.62 |
| FL^a’,b’^ [kN] | 2.32 | 3.26 | 8.91 | 3.56 | **2.48** | **0.75** | **1.05** | **1.20** |
| Stiffness^a’,b’^ [kN/mm] | 2.19 | 2.62 | 8.14 | 3.72 | **2.73** | **0.74** | **1.24** | **1.36** |
| **Cortical** |  |  |  |  |  |  |  |  |
| Ct.BMD^b^ [mg HA/cm^3^] | **0.35** | **0.37** | **0.69** | **0.70** | **0.37** | **0.58** | **0.43** | **0.73** |
| Ct.Ar^a^ [mm^2^] | **1.84** | **1.99** | **0.71** | **2.31** | 0.83 | 2.01 | 1.20 | 1.83 |
| Ct.Th [mm] | 2.00 | 1.99 | 1.03 | 2.72 | 1.02 | 2.12 | 1.44 | 1.56 |
| Ct.Po [%] | 32.11 | 21.65 | 19.85 | 24.86 | 23.26 | 21.24 | 21.01 | 16.39 |
| **Trabecular** |  |  |  |  |  |  |  |  |
| Tb.BMD [mg HA/cm^3^] | 0.94 | 1.03 | 0.77 | 0.61 | 1.22 | 1.47 | 0.75 | 1.54 |
| Tb.Ar^a,b^ [mm^2^] | 0.48 | 0.51 | 0.32 | 0.70 | **0.21** | **0.61** | **0.35** | **0.31** |
| Tb.BV/TV [%] | 1.99 | 1.31 | 2.97 | 1.38 | 1.37 | 1.32 | 0.71 | 1.02 |
| Tb.N [1/mm] | 2.10 | 1.76 | 2.02 | 3.02 | 2.60 | 2.49 | 3.37 | 2.70 |
| Tb.Sp [mm] | 1.32 | 0.88 | 1.64 | 1.58 | 1.34 | 1.63 | 1.63 | 1.84 |
| Tb.Th^a’^ [mm] | 1.15 | 1.01 | 1.29 | 1.43 | **1.71** | **1.70** | **0.52** | **2.19** |

**^a^*p*<0.05** from Kruskal-Wallis test comparing median precision values across position in study block

**^a’^*p*<0.05** from Kruskal-Wallis test following false discovery rate adjustment with Benjamini-Hochberg procedure

**^b^*p*<0.05** from Levene’s test comparing variance in precision values across position in study block

**^b’^*p*<0.05** from Levene’s test following false discovery rate adjustment with Benjamini-Hochberg procedure

Supplemental Table 4: Precision results (RMS-CV%) for registered data stratified by BMI categories.

|  | **Distal Radius (n=40)** | | | **Distal Tibia (n=44)** | | |
| --- | --- | --- | --- | --- | --- | --- |
| Body mass index (BMI) | BMI<25 | 25≤BMI<30 | BMI≥30 | BMI<25 | 25≤BMI<30 | BMI≥30 |
|  | n=10 | n=16 | n=14 | n=11 | n=18 | n=15 |
| **Total** |  |  |  |  |  |  |
| Tt.BMD [mg HA/cm^3^] | 0.66 | 0.87 | 0.71 | 0.24 | 0.20 | 0.71 |
| FL [kN] | 4.43 | 3.41 | 7.41 | 1.62 | 1.60 | 1.45 |
| Stiffness [kN/mm] | 4.91 | 3.28 | 6.32 | 1.90 | 1.66 | 1.65 |
| **Cortical** |  |  |  |  |  |  |
| Ct.BMD [mg HA/cm^3^] | 0.52 | 0.56 | 0.56 | 0.50 | 0.52 | 0.57 |
| Ct.Ar [mm^2^] | 0.84 | 2.25 | 1.67 | 1.57 | 1.59 | 1.36 |
| Ct.Th [mm] | 1.57 | 2.10 | 2.12 | 1.64 | 1.45 | 1.65 |
| Ct.Po [%] | 24.71 | 20.27 | 28.85 | 22.78 | 23.29 | 15.80 |
| **Trabecular** |  |  |  |  |  |  |
| Tb.BMD [mg HA/cm^3^] | 1.05 | 0.83 | 0.74 | 1.36 | 0.78 | 1.60 |
| Tb.Ar [mm^2^] | 0.27 | 0.55 | 0.59 | 0.38 | 0.40 | 0.41 |
| Tb.BV/TV [%] | 2.67 | 1.81 | 1.80 | 1.15 | 0.90 | 1.36 |
| Tb.N [1/mm] | 1.66 | 2.60 | 2.15 | 2.11 | 2.70 | 3.37 |
| Tb.Sp [mm] | 1.59 | 1.38 | 1.22 | 1.32 | 1.41 | 1.96 |
| Tb.Th [mm] | 1.50 | 1.16 | 1.06 | 1.80 | 1.23 | 1.82 |

**Note:** All p>0.05 from Kruskal-Wallis and Levene’s tests comparing variance in precision values across BMI categories following false discovery rate correction

Supplemental Table 5: Precision results (RMS-CV%) for registered data stratified by age categories.

|  | **Distal Radius (n=40)** | | | | **Distal Tibia (n=44)** | | | |
| --- | --- | --- | --- | --- | --- | --- | --- | --- |
| Age group | <30 | 30-49 | 50-69 | 70+ | <30 | 30-49 | 50-69 | 70+ |
|  | n=13 | n=12 | n=11 | n=4 | n=14 | n=13 | n=13 | n=4 |
| **Total** |  |  |  |  |  |  |  |  |
| Tt.BMD [mg HA/cm^3^] | 0.78 | 0.71 | 0.86 | 0.60 | 0.51 | 0.27 | 0.55 | 0.29 |
| FL [kN] | 3.90 | 7.44 | 4.60 | 3.77 | 1.09 | 1.71 | 1.92 | 0.96 |
| Stiffness [kN/mm] | 4.41 | 6.56 | 3.83 | 3.20 | 1.26 | 1.74 | 2.23 | 1.02 |
| **Cortical** |  |  |  |  |  |  |  |  |
| Ct.BMD [mg HA/cm^3^] | 0.63 | 0.49 | 0.50 | 0.61 | 0.49 | 0.38 | 0.54 | 0.94 |
| Ct.Ar [mm^2^] | 2.09 | 1.18 | 1.65 | 2.45 | 1.42 | 1.13 | 1.44 | 2.69 |
| Ct.Th [mm] | 2.32 | 1.70 | 1.68 | 2.37 | 1.78 | 1.16 | 1.60 | 1.81 |
| Ct.Po [%] | 18.36 | 32.78 | 22.91 | 17.91 | 11.69 | 20.85 | 28.65 | 16.34 |
| **Trabecular** |  |  |  |  |  |  |  |  |
| Tb.BMD [mg HA/cm^3^] | 0.53 | 0.95 | 1.09 | 0.70 | 1.07 | 0.85 | 1.58 | 1.71 |
| Tb.Ar [mm^2^] | 0.67 | 0.33 | 0.48 | 0.45 | 0.42 | 0.35 | 0.41 | 0.41 |
| Tb.BV/TV [%] | 2.29 | 2.16 | 1.87 | 1.19 | 0.99 | 1.03 | 1.47 | 0.63 |
| Tb.N [1/mm] | 2.96 | 1.87 | 1.92 | 1.02 | 2.72 | 2.38 | 3.54 | 1.61 |
| Tb.Sp [mm] | 1.82 | 1.11 | 1.24 | 0.70 | 1.69 | 1.35 | 1.71 | 1.66 |
| Tb.Th [mm] | 1.17 | 1.41 | 0.81 | 1.67 | 1.07 | 1.17 | 2.02 | 2.53 |

**Note:** All p>0.05 from Kruskal-Wallis and Levene’s tests comparing variance in precision values across age categories following false discovery rate correction

Supplemental Table 6: Precision results and differences in RMS-CV% for registered data with and without exclusion for scans with motion grades greater than 3.

| **Parameter** | **Distal Radius (n=40)** | | | **Distal Tibia (n=44)** | | |
| --- | --- | --- | --- | --- | --- | --- |
|  | **Full Cohort (All Motion Grades)**  **RMS-CV%**  **(n=45)** | **With Exclusions (Only Motion Grades 1-3)**  **RMS-CV%**  **(n=40)** | **%∆** | **Full Cohort (All Motion Grades)**  **RMS-CV%**  **(n=45)** | **With Exclusions (Only Motion Grades 1-3)**  **RMS-CV%**  **(n=44)** | **%∆** |
| **Total** |  |  |  |  |  |  |
| Tt.BMD [mg HA/cm^3^] | 0.80 | 0.77 | -3.75% | 0.45 | 0.45 | 0.00% |
| FL [kN] | 5.89 | 5.37 | -8.83% | 1.54 | 1.55 | +0.65% |
| Stiffness [kN/mm] | 5.48 | 4.93 | -10.04% | 1.70 | 1.72 | +1.18% |
| **Cortical** |  |  |  |  |  |  |
| Ct.BMD [mg HA/cm^3^] | 0.73 | 0.55 | -24.66% | 0.58 | 0.53 | -8.62% |
| Ct.Ar [mm^2^] | 1.79 | 1.79 | 0.00% | 1.72 | 1.51 | -12.21% |
| Ct.Th [mm] | 2.01 | 1.99 | -0.99% | 1.79 | 1.57 | -12.29% |
| Ct.Po [%] | 26.45 | 24.66 | -6.77% | 20.96 | 20.90 | -0.29% |
| **Trabecular** |  |  |  |  |  |  |
| Tb.BMD [mg HA/cm^3^] | 0.94 | 0.86 | -8.51% | 1.30 | 1.26 | -3.08% |
| Tb.Ar [mm^2^] | 0.50 | 0.51 | +2.00% | 0.46 | 0.40 | -13.04% |
| Tb.BV/TV [%] | 2.16 | 2.05 | -5.09% | 1.33 | 1.14 | -14.29% |
| Tb.N [1/mm] | 2.58 | 2.24 | -13.18% | 2.90 | 2.82 | -2.76% |
| Tb.Sp [mm] | 1.82 | 1.38 | -24.18% | 1.79 | 1.60 | -10.61% |
| Tb.Th [mm] | 1.47 | 1.22 | -17.01% | 1.67 | 1.60 | -4.19% |

**Note:** Cohort without exclusions (n=45) used as reference
